# A comparison of the effects of ENTONOX inhalation and spinal anesthesia on labor pain reduction and apgar score in vaginal delivery: a clinical trial study

**DOI:** 10.1051/bmdcn/2018080317

**Published:** 2018-08-24

**Authors:** Samira Foji, Manijeh Yousefi Moghadam, Hosein TabasiAsl, Milad Nazarzadeh, Hamid Salehiniya

**Affiliations:** 1 School of Nursing and Midwifery, Sabzevar University of Medical Sciences, Sabzevar, Iran,PHD candidate in nursing, school of nursing and Midwifery, Golestan university of medical sciences Golestan Iran; 2 School of Medicine, Sabzevar University of Medical Sciences Sabzevar Iran; 3 Sabzevar University of Medical Sciences Sabzevar Iran; 4 Social Medicine Department, School of Medicine, Sabzevar University of Medical Sciences Sabzevar Iran; 5 Zabol University of Medical Sciences Zabol Iran; 6 Department of Epidemiology and Biostatistics, Tehran University of medical sciences Tehran Iran

**Keywords:** Delivery, ENTONOX, Spinal anesthesia, Labor pain

## Abstract

Introduction: The purpose of the present study was to compare the effect of ENTONOX inhalation and spinal injection on the reduction of labor pain, the Apgar score of the neonates, and their side effects on new-born children and pregnant women.

Material and Methods: The present clinical trial study is conducted among the pregnant women in the maternity ward of a child delivery hospital in Iran. All Participants were divided in two groups ENTONOX Inhalation and Spinal Anesthesia. Visual Analogue Scale (VAS) was implemented to measure the pain level experienced by the participants during the procedure. Moreover, the Apgar scale was used to measure the general physical health of the neonates in both groups. In addition, the participants receiving ENTONOX were asked to report the side effects they underwent during gas inhalation. However, the participants in the spinal anesthesia were checked three times. Statistical analysis was performed using SPSS version 22.

Results: The findings showed that the spinal anesthesia technique was significantly more effective than gas inhalation in that it reduced as much as 3 points more than did the inhalation (*P*-value: 0.001). Moreover, the comparison of the mean Apgar scores showed that the mean Apgar score of the neonates of spinal anesthesia mothers was 0.36 point lower than that of the neonates in the gas inhalation group. However, this difference was not statistically significant at *P*- value = 0.06.

Conclusions: the result of the present study indicated that spinal anesthesia was more effective than ENTONOX inhalation in reducing the labor pain.

## 1. Introduction

Labor pain is found to be one of the most agonizing types of pain ever experienced, described as being either severe or intolerable by 35 to 58 percent of women in labor[1, 2]. There are a number of different (pharmacological) medicinal and non-medicinal methods available for the management of the labor pain [3, 4]. There are different techniques to provided labor analgesia including intravenous analgesics, inhalation therapy, acupuncture, and local anesthesia [5]. If anesthesia is employed, it should be safe both for the mother and fetus [6].

In anesthesia technique, one of the most frequently used analgesic agents is ENTONOX.

ENTONOX® is a mix of Nitrous oxide 50% and oxygen 50% [7]. It is a very effective analgesic agent with rapid onset and offset characteristics. Although ENTROX is a non-inflammable, tasteless, odorless and colorless gas [8], it shown to be associated with some side effects such as excessive drowsiness, dizziness or light headedness, nausea and vomiting, dry mouth, buzzing in the ears, rarely “pins and needles” or numbness, dreams, a hazymemory of labor, feelings of claustrophobia with use of mask [9, 10].

Among local analgesia methods, spinal block has been employed more frequently to reduce the labor pain for years in that it enjoys some advantages over other local analgesia methods including a faster analgesia onset time, a higher analgesia quality, a lower dosage of drug needed and a higher rate of success. (Epidural triggers analgesia sooner) [11, 12]. However, there are some disadvantages associated with the employment of spinal block such as headache, drop in blood pressure, and transient backaches [13].

The efficacy of the anesthesia technique employed (applied) greatly depends on the condition of the mother, and more importantly, on the general condition of the newborn [14, 15]. The best parameter used to assess the immediate condition of the baby after delivery is the Apgar score [16, 17]. It is the first simple and repeatable method performed on the newborn baby in the delivery or birthing room a make a quick and accurate assessment of the physical health condition of the newborn baby immediately after delivery so that extra medical and emergency care can be provided if needed. [1, 18, 19]

Therefore, the aim of the present study was to compare two methods of analgesia with spinal and Entonox in reducing labor pain, mother complications and neonatal Apgar.

## 2. Material and Methods

The present clinical trial was conducted to compare the relative efficacy (effectiveness) of two analgesia techniques, ENTONOX and spinal block, in reducing the pain labor in normal delivery, and to examine the effects of these two analgesia techniques on APGAR score of newborn children, and on pregnant women who hospitalized in Sabzevar (northeastern province of Iran) in 2016. The population of the study included all pregnant women admitted to Hospital for a normal labor delivery, who indicated their formal consent to participate voluntarily in the study. The participants were not forced to choose one of the two analgesia techniques. They were absolutely free to choose the analgesia technique which preferred for their normal delivery. If the subjects met the inclusion criteria for the study [20, 21], they were put into one of the experimental groups, spinal block or ENTONOX gas based on the analgesia technique they choose. The ethical approval of the present study was issued by the Ethical Committee of Sabsevar Medical University (ir.medsab.rec. 1394.9).

## 3. The Inclusion and Exclusion Criteria

The following inclusion criteria are characteristics that the prospective subjects must have if they are to be included in the study, and allowed to participate in the study: (1) mothers who were considered candidates for a normal delivery with one of the analgesia methods of ENTONOX gas or Spinal block, (2) mothers who were in their active stage of labor (with a cervical dilation of 3 to 4 centimeters, and a cervical effacement of 40 to 50%), (3) mothers who were in their pregnancy term (their period of pregnancy was from 37 to 42 weeks), (4) cephalic presentation. (based on the latest Ultrasonography) [22]

Moreover, in the present study, the following exclusion criteria were employed to ensure the safety of the participants. Mothers were excluded from the study if (1) they showed no consent to take part in the study. (2) There was a presence of infection in the injection area, (3) they showed coagulation disorders, (4) they showed Cranial pressure symptoms, (5) they showed counterindicators for the use of ENTONOX gas including an acute severe headache, dyspnea, asthma, emphysema, Ileus, or chest trauma associated with dyspnea [23, 24].

### 3.1. Variable and Measurement Methods

For the purpose of the study, the necessary demographic data related to the participants were collected using appropriate questionnaires and forms. Moreover, the severity of the labor pain experienced by the participants was implemented by the Visual Analogue Scale [25, 26]. This scale measures the severity of pain in 10 levels, with 10 showing the highest level of pain severity. The mother participants in ENTONOX group were asked to rate the level of pain they experienced before gas inhalation, choosing a number between 0 and 10 as described by VAS. After four ENTOXON inhalations, they were required to rate the pain they suffered again. The participants in the spinal group were asked to measure the level of pain they experienced before and five minutes after the reception of a spinal injection of narcotic, using the ten levels of pain as described in VAS. In addition, the general physical health of the neonates in both groups were assessed immediately after birth using the Apgar checklist. (form) [27, 28]

The followings were considered to be the side effects of ENTONOX inhalation: lightheadedness, nausea, vomiting, tiredness, dry mouth, a feeling of tingling in fingers, an increase in sleepiness, and tinnitus in the ear while the side effects associated with the administration of spinal injection were vomiting, hypertension, itching, anesthesia in feet and legs, headache, urinary retention and vision problems including a blurred vision and diplopia or double vision, which are in agreement with the previous studies in this field [16, 29, 30]. Moreover, the participants in the ENTONOX group were asked for the adverse side effects during the inhalation session while the participants in the spinal injection group were examined for the negative side effects of the injection in three time periods (immediately after the implementation of spinal injection, 12 hours after delivery, and before leaving maternity hospital).

### 3.2. Intervention Procedures

In this study, ENTONOX was used to induce anesthesia in the participants in the Inhalation group. On their arrival at the maternity ward of the Hospitals, the participants had regular access to ENTONOX inhalation system (instruments). They were instructed to inhale the anesthesia gas as soon as they experienced the labor pain associated with uterine contractions, and to stop inhalation once the pain is relieved. Due to the fact that the pain relief effect of fentanyl and pethidine agents is temporary and transient (short-lived), the participants in the spinal injection group received these agents only when they were in the active phase of delivery, that is, the cervical dilation for nulliparous women, mothers who had not given birth before, was 6 centimeters, and for multiparous women, those who had given birth previously, 5 centimeters. The anesthesia was induced in the participants in the spinal injection through giving them 20 milligrams of fentanyl along with 15 milligrams of pethidine. For the injections, the anesthesia specialist used a 27 needle with pen-like point to give the participant and injection in either L3-L4 or L4-L5 landmarks (interspaces).

Ethical consideration: This study is based on the ethics of medical research on human subject and Helsinki statement and written consents were obtained from all patients. This trial is registered with irct.ir, number IRCT2016040316148N3.

### 3.3. Statistical Tests and Analyses

According to predefined calculation of sample size with statistical power of 80% in the protocol, we allocated 60 patients to group Entonox gas and 60 patients to group spinals. The independent t-test and chi-square test were used as the primary test of an overall difference between two methods across considered variables. Because of non-randomized nature of this study, multivariate regression model was necessary for adjustment of confounding effects. We used multiple linear regression models for modelling of considered independent variables on the Apgar score and pain after intervention Forced entry method was used for variable selection and adjusted regression coefficient with 95% confidence intervals (CI 95%) was used as desired measure of association in this study. Assumptions of the model were checked using scatter plot (linear relationship), histogram and Q-Q plot (checking normality) and variance inflation factor (VIF) for checking of multicollinearity in the model. All statistical analysis was performed using SPSS version 19.

## 4. Result

In this study, a total number of 120 pregnant mothers who were candidates for normal labor were assigned to two experimental groups of anesthesia (spinal injection *vs*. ENTONOX inhalation), with an average age of 26.07 (with a SD of 5.41) and 25.98 (with a SD of 4.71), respectively. In the Spinal group, the mothers had an average weight of 72.50 while those in the gas inhalation group had an average weight of 72.11 with a standard deviation of 6.51. The Spinal group mothers had normal cephalic presentations while, in the ENTONOX group, 56 percent of them had a normal cephalic presentation, with a bridge of 4 (%6.7). The two experimental groups were normally distributed in terms the above mentioned variables (Table 1).

**Table 1:** Characteristics of the variables under study.

Variables	Gas	Spinal	*P*-value
	(n = 60)	(n = 60)	
	Mean (SD)	Mean (SD)	
Mother Age	25.98 (4.71)	26.07 (5.41)	0.92
Mother Weight	72.11 (6.51)	72.50 (10.13	0.80
Apgar	9.15 (.065)	8.76 (1.19)	0.03
Baby Head size	34.38 (1.42)	34.45 (1.37)	0.81
Dilatation	6.48 (1.17)	5.60 (1.63)	0.001
Effacement	65.50(12.23)	60.83 (14.29	0.05
Pain before (in gas group)	9.16 (1.52)	9.46 (1.37)	0.25
Pain before (in spinal group)	4.38 (2.63)	1.65 (1.99)	0.00
variables	n (%)	n (%)	*P*-value
**Gender of baby**			
Male	29 (48.3)	32 (53.3)	
Female	31 (51.7)	28 (46.7)	0.58
**Location**			
city	40 (66.7)	45 (75)	
village	20 (33.3)	15 (25)	0.31
**Addiction of mother**			
No	60 (0)	58 (96.7)	
Yes	0 (0)	2 (3.3)	0.42
**Delivery times**			
Nulliparous	35 (58.3)	34 (56.7)	
Multigravida	25 (41.7)	25 (41.7)	0.60
**History of before delivery**			
first delivery	36 (60)	34 (56.7)	
Natural childbirth	24 (40)	25 (41.7)	0.85
Caesarean	0 (0)	1 (1.7)	
**Use of analgesia in the previous delivery**			
first delivery	36 (60)	36 (60)	
Yes	19 (31.7)	8 (13.3)	0.06
No	5 (8.3)	16 (26.7)	
**Previous delivery anesthesia**			
first delivery	39 (65)	51 (85)	
gas	18 (30)	6 (10)	0.03
spinal	2 (3.3)	2 (3.3)	
**Fetal positions**			
sefalic	56 (93.3)	60 (0)	0.11
brich	4(6.7)	0 (0)	

The findings of the multiple regression analyses performed on the data related to the two experimental groups demonstrated that, after the implementation of anesthesia, pain level among subjects in the spinal injection group measured by VAS, was 3 points lower than that experienced by the subjects in the other group. The results of the analyses showed that the difference between the pain levels experienced by the subjects in the two groups was statistically significant at *P*-value of *p <* 0.001 (Table 2).

**Table 2:** Multiple regression analysis of the relationship of the pain level after anesthesia with independent variables.

Variables	Unstandardizedcoefficients (95% CI)	Standardized Coefficients	*P*-value
Method of anesthesia (gas or spinal)	-3.01 (-3.93 to -2.09)	-0.56	*p* < 0.001
Age of mather	-.003 (-0.10 to 0.09)	-0.005	0.95
Weight of mother	-0.01 (-0.06 to 0.03)	-0.04	0.58
Addiction	-1.95 (-5.2 to 1.3)	-0.09	0.24
Series of labor	-1.2 (-4.23 to 1.76)	-0.23	0.41
Kind last labor	0.67 (-2.12 to3.47)	0.12	0.63
Use analgesia last labor	0.38 (-0.76 to 1.52)	0.11	0.50
Dilitation	-0.06 (-0.55 to 0.41)	-0.03	0.77
Efasman	-0.005 (-0.05 to 0.04)	-0.02	0.85
Situation fetus	-1.36 (-3.94 to 1.21)	-0.09	0.29
Head size	-0.03 (-0.34 to 0.27)	-0.01	0.81
Pain before	0.38 (0.06 to 0.69)	0.20	0.01

R square: .085, Adjusted R square:-0.08, ANOVA: .528

Moreover, the results of the linear regression analysis showed that, compared to the use of ENTONOX, the implementation of spinal injection reduced the Apgar measure of the neonates as much as 0.36, with all other variables being hold constant. However, the difference observed in the effect of anesthesia technique on the reduction of Apgar measures of the two groups was not statistically significant at *P*-value of 0.06 (Table 3).

**Table 3:** Multiple regression alysis of the relationship of the Apgar level after anesthesia with independent variables.

Variables	Unstandardized coefficients (95% CI)	Standardized Coefficients	*P*-value
Method of anesthesia (spinal or gas)	-0.36 (-0.73 to 0.01)	- 0.18	0.06
Age of mother	-0.01 (-0.04 to 0.02)	-0.06	0.5
Weight of mother	0.01 (-0.01 to 0.03)	0.09	0.3
Addiction (yes vs. no)	0.3 (-1.05 to 1.75)	0.04	0.6
Dilitition	0.003 (-0.20 to 0.19)	-0.004	0.97
Efasman	-0.003 (-0.02 to 0.01)	-0.04	0.78
Situation fetus	0.24 (-0.82 to 1.31)	0.54	0.65
Head size	-0.037 (-0.16 to 0.09)	-0.05	0.09
Pain before	-0.11 (-0.25 to 0.01)	-1.77	0.14

R square: .060, Adjusted R square: -.008, ANOVA: .528

The examination of the subjects for the possible side effects of Spinal technique in three time intervals (immediately after the injection, 12 hours after the injection, and when mothers were leaving the hospital) showed that the implementation of this technique to induce anesthesia was associated with the following important side effects:

Immediately after receiving spinal injection, the subjects experienced nausea 25 (42.4), vomiting 14 (23.3), hypotension 11 (18.3), itching 29(48.3), and anesthesia 30 (50). Twelve hours after the injection, they suffered itching 10 (16.7), anesthesia 6 (10), headache 8 (13.3), and Urinary retention 1 (1.7). When they were discharged the hospital, they experienced hypotension 1 (1.7), itching 6 (10), and anesthesia 5 (8.3) (Table 4).

**Table 4:** Complications of spinal anesthesia in mothers at three different times.

Complications	0 min	12 h	Discharge time
Nausa	N (%)	N (%)	N (%)
No	34 (57.6)		
Yes	25 (42.4)		
**Vomiting**			
No	46 (76.7)		
Yes	14 (23.3)		
**Hypotention**			
No	49 (81.7)		59 (98.3)
Yes	11 (18.3)		1 (1.7)
**Itching**			
No	31 (51.7)	50 (83.3)	54 (90)
Yes	29 (48.3)	10 (16.7)	6 (10)
**Numb**			
No	30 (50)	54 (90)	55 (91.7)
Yes	30 (50)	6 (10)	5 (8.3)
**Headache**			
No		52 (86.7)	55 (91.7)
Yes		8 (13.3)	5 (8.3)
**Urinary retention**			
No		59 (98.3)	60 (100)
Yes		1 (1.7)	0 (0)
**Diplopy**			
No		60 (100)	60 (100)
Yes		0 (0)	0 (0)
**Impaired vision**			
No		60 (100)	60 (100)
Yes		0 (0)	0 (0)
**Photophoby**			
No			60 (100)
Yes			0 (0)

The examination of the subjects in the other experimental group showed that the use of ENTONOX as a technique to produce anesthesia was associated with the following side effects: lightheadedness 38 (63.3), nausea 8 (13.3), vomiting 1 (1.7), tiredness 17 (28.3), a dry mouth 43 (17.7), tingling of the fingers 3 (5), an increase in sleepiness 41 (68.3), and tinnitus 1 (1.7) (Table 5).

**Table 5:** Complications of gas anesthesia in mothers at three different times.

Complications	N (%)
**Light headedness**	
No	22 (36.7)
Yes	38 (63.3)
**Nausa**	
No	52 (86.7)
Yes	8 (13.3)
**Vomiting**	
No	59 (98.3)
Yes	1 (1.7)
**Increased fatigue**	
No	43 (71.7)
Yes	17 (28.3)
**Xerostomia**	
No	17 (28.3)
Yes	43 (71.7)
**Tingling of the fingers**	
No	57 (95)
Yes	3 (5)
**Increased sleep**	
No	19 (31.7)
Yes	41 (68.3)
**Tinnitus**	
No	59 (98.3)
Yes	1 (1.7)

## 5. Discussion

An exhaustive review of the relevant literature showed that there were not any similar studies conducted before to compare the side effects, and the efficacy of the implementation of ENTONOX and Spinal injection on labor pain reduction and the Apgar measures of neonates. Hence, the findings of the present study were compared with the findings obtained in studies dealing with other similar anesthesia induction techniques.

The present study was an attempt to compare the relative efficacy and reliability of spinal injection and ENTONOX inhalation as two techniques for inducing anesthesia in mothers who had a normal labor. The comparison of the pain levels experienced by the subjects in the two experimental groups as measured by VAS, before and after the implementation of the corresponding anesthesia techniques revealed that, compared to ENTONOX inhalation, spinal injection was a meaningfully more effective method to reduce the severity of labor pain in that it triggered a reduction in the pain experienced which was as much as three points larger than that produced by ENTONOX inhalation. This finding could be accounted for the fact that in spinal technique, the pain-relief agent (medicine) is injected into the spinal space where there is more systemic absorption of the agent.

In Teimori *et al.* study on labor pain reduction through ENTONOX inhalation, they observed that the subjects receiving ENTONOX experienced significantly less pain compared to those who received only oxygen. The findings of the present study is in line with the findings obtained by Teimori [31].

In another study, Mazul-Sunko showed that the local and spinal injection of an anesthetic agent or opium had a significant effect on labor pain reduction, with a fast onset and fewest side effects [32]. His findings are compatible with those obtained in the present study.

In their study on the safety and reliability of the injection of a single dose of anesthetic agent during normal labor, Minty R. G. *et al.* revealed that, compared to epidural techniques, spinal anesthesia was more reliable and effective in reducing labor pain [33].

Apgar is a systematic way to assess the general health condition of the neonate immediately after birth so that babies who need medical care can have more survival chances. A low Apgar measure can be due to a variety of factors one of which is the drugs (medicine) taken by the mother during labor (normal or Caesarean) [34].

The comparison of the Apgar measures of the neonates in the two experimental groups in the present study showed that, on average, the spinal injection of anesthetic agent was associated with a reduction of the mean Apgar measure of the neonates of this group, which was as much as 0.36 larger than that created by gas inhalation. Since the examination of the cumulative frequency distribution of the neonates revealed that 4.2 % of the neonates had an Apgar measure of less than 7, it could not be easily explained why the use of spinal injection was found to be associated with a larger reduction in the mean. Apgar measure of the neonates of the mothers in that group However, a *P*-value of 0.06 can be of clinical importance. In the study done by Naddoni on the effects of ENTONOX inhalation, it was shown that the inhalation of this gas had no effect on the Apgar measures of the neonates in the first and the fifth minutes after birth [35]. In a study with the topic of comparison of adverse effects of Spinal injection and general anesthesia induction techniques on neonates of 175 pregnant women who had active Caesarean, Mancuso *et al.* showed that the implementation of spinal technique was associated with a higher mean Apgar measure. Moreover, the babies of the mothers who received spinal injection needed less ventilation as compared with those of the mothers in the other group. The findings of this study are not congruent with those observed in the present study [36].

The findings of another study conducted to compare the effect of general anesthesia induction and Spinal-epidural injection on the Apgar measures of the neonates suggested that the mean Apgar measure for the neonates of the mothers who received an epidural-spinal combined injection was meaningfully higher than those for the neonates of the mothers in the general anesthesia group. The findings indicated that the spinal-epidural injection was safer for the mothers and the neonates compared to the general anesthesia induction technique [37]. This finding can be explained by the effect of transient sedation secondary to the general anesthetic agents.

Contrary to the studies mentioned so far, in their meta-analysis devoted to the comparison of different anesthesia techniques, Reynolds and Seed observed that the PH level of the blood in the neonates of the participants receiving Spinal injection for pain relief was lower as compared to that for the participants who had general anesthesia and epidural techniques. It cannot be concluded, therefore, that the spinal technique is safer for the participants compared to the other two techniques implemented in the study [38].

In other study, Hassan SarhabHaider *et al.* compared general anesthesia (GA) and Spinal injection as pain control techniques. The results of their study showed that the Apgar measures of the neonates of the mothers in general anesthesia group in the first minute after birth were meaningfully lower than those of the neonates of the mothers in the spinal injection group. However, such a statistically meaningful difference was not observed between the Apgar measures of the two groups in the fifth minute after birth. However, it must be noted that the mother participants in this study had requested a C-section (caesarean) to deliver their babies.

In another study, Krzysztof and Susilo Chandra focused on the satisfaction level of Indonesian mothers with Spinal anesthesia for the management of their labor pain. In this study, some 62 women with singleton pregnancy and term, with 45 of them being nulliparous women and 17 multiparous ones. All the participants received a spinal injection containing a mix of 2.5 milligram of Bupivacaine, 0.25 milligram of Morphine, and 45 milligram clonidine. In this study, the satisfaction rate of the participants, the pain term, pain relief, and the side effects of the spinal injection received were examined. On the whole, the participants showed a high rate of satisfaction with the efficacy of spinal anesthesia for the elimination of labor pain, with 50 (81%) of them showed great satisfaction and 7 (11%) of them fairly satisfied with the technique employed. In addition, the majority of the participants, nearly 79%, stated that in their future pregnancies they will choose again the spinal anesthesia to control their labor pain [14].

In the present study, the examination of the participants undergoing ENTONOX inhalation for the possible side effects showed that the most commonly experienced physical side effects were: 0.38 lightheadedness, 0.43 a dry mouth, and 0.41 sleepiness. In a similar study, Naddoni D. B. *et al.* showed that the subjects receiving ENTONOX experienced meaningfully more adverse side effects compared to those receiving oxygen. More specifically, 61.63 of the participants felt sleepiness, 9.30% had a dry mouth, 50% had lethargy, 26.74% had blurred vision, 30.23% felt tingling in their limbs, 36% faced vomiting, 23.26% had headache, and finally 31.40% had an uncomfortable feeling [35].

In the study done by Najafiyan, the most commonly observed side effect after receiving ENTONOX was lethargy (41%) while the least frequently observed one was uncomfortable feeling (12%), with other side effects coming in between including vertigo (20%), vomiting (11%), and a dry mouth (31%). Similar to other studies, the findings of this study showed that the inhalation of ENTONOX was associated with more adverse side effects compared to spinal anesthesia [39].

The negative side effects frequently associated with spinal anesthesia are found to be headache, Hypotension, and backache (Hyderally, 2002). In the same vein, in our study, the examination of the participant undergoing spinal anesthesia in three time intervals, that is, immediately after the injection, 12 hours after injection, and at the time of leaving hospital, showed that they generally suffered vomiting, nausea, headache, hypotension, and itching.

In another study, Ghodsi investigated the effects of spinal anesthesia, induced by injecting 10 micrograms of fentalyn along with 1cc of Morphine, on normal delivery (natural child delivery). The results obtained in this study showed that the rate of use of vacuum extractor in the experimental group was meaningfully higher than that in the control group, an observation which may be accounted for by the interferences (disturbances) caused in the muscle contractions. Moreover, the results suggested that the side effects triggered by the implementation of spinal anesthesia were minimal, with no need for medications. The results of all the 19 research studies done so far on the side effects associated with spinal anesthesia have indicated that the most frequently observed side effect is itching, and that 10 to 40 percent of the mothers receiving spinal injection to control their labor pain were faced with a Hypotension. Despite the side effects that follow spinal anesthesia, 95% of mothers express their satisfaction with the efficacy of this pain relief technique [40].

The most important limitation of the present study which endanger the validity and generalizability of the findings of the present study is the fact that the participants were not selected and assigned to the two experimental groups on a random basis. In other words, the participants in this study selected the pain relief technique they preferred to receive out of their volition.

## 6. Conclusions

Based on the results obtained in this study, it seems that the spinal anesthesia is preferable to ENTONOX inhalation in that it is more effective in reducing the labor pain compared to ENTONOX inhalation, and causes fewer negative side effects on both the mother and the neonate.

## Conflicts of interest

The authors declare no conflict of interest.
